# Taking a paradoxical perspective on bedtime procrastination

**DOI:** 10.3389/fpsyg.2023.1096617

**Published:** 2023-02-08

**Authors:** Nanxi Yan

**Affiliations:** ^1^Department of Psychology, Faculty of Behavioural and Social Sciences, University of Groningen, Groningen, Netherlands; ^2^Department of HRM and OB, Faculty of Economics and Business, University of Groningen, Groningen, Netherlands

**Keywords:** bedtime procrastination, paradox theory, paradox mindset, sleep, leisure time, leisure activities, wellbeing

## 1. Introduction

Societal change driven by technological advancement and the blurring of the lines between work and leisure has resulted in more and more individuals not getting sufficient sleep during workdays, despite knowing that sleep is critical for mental, emotional, and physical health (Mukherjee et al., 2015). One behavior that gives rise to sleep deprivation is *bedtime procrastination* (Kroese et al., 2014). Bedtime procrastination is the decision to go to bed later than intended in the absence of external reasons, such as a night shift or family responsibilities (Kroese et al., 2014). This is a widespread phenomenon in both Western countries such as the United States and the Netherlands (Kroese et al., 2014, 2016), and many Asian countries (Kadzikowska-Wrzosek, 2020). As a relatively new concept, current research on bedtime procrastination mainly focuses on antecedent phenomena (e.g., chronotype, Kuhnel et al., 2018) and outcomes (e.g., daytime fatigue, Hill et al., 2022). Fewer studies, however, have examined the process of bedtime procrastination. This opinion paper aims to bridge this gap by shedding light on how a paradoxical perspective can shape the process of dealing with bedtime procrastination.

## 2. The paradoxical tension between leisure and health pursuits in bedtime procrastination

Paradoxes are “contradictory, yet interrelated elements that exist simultaneously and persist over time” (Smith and Lewis, 2011, p. 382) [sic]. At the core of the paradox perspective is the notion that tension is embedded and, thus, embracing contradictory and interrelated demands simultaneously is vital for success (Smith and Lewis, 2011). This perspective has been used to examine a wide range of phenomena, such as managing artificial intelligence in organizations (Raisch and Krakowski, 2021) and competing for organizational identities (Ashforth and Reingen, 2014). Nevertheless, research adopting the paradox perspective often focuses on tensions operating at the organizational level; it is relatively less studied in terms of individual behavior (Zheng et al., 2018), including health behavior.

With regard to bedtime procrastination, there are two distinct and sometimes competing demands: leisure and health. Individuals have leisure needs, such as wanting to detach, recover, or socialize, that are important to their wellbeing (Kuykendall et al., 2015). However, engaging in leisure activities during late-night hours can be incompatible with health goals. This is because delaying sleeping for leisure activities can trigger *cognitive dissonance* (Festinger, 1957), wherein a discrepancy between “what I want to do” (e.g., surfing the internet, hedonic values) and “what I am expected to do” (e.g., sleep hygiene, sufficient recovery) is experienced. This form of attitude-behavior disconnect can cause negative emotions in individuals. The tension between leisure and health can also be explained by resource (i.e., time) conflicts (Riediger and Freund, 2004), in which individuals want to satisfy both hedonic or leisure needs and health needs at the same time, but one comes at the expense of the other, and they compete for the same time resource.

## 3. The paradox mindset plays a role in dealing with the tension

To deal with tension, research suggests that individuals first attempt to understand or appraise the experience before employing coping strategies (Lazarus, 1991). A *mindset* refers to a framework or lens that can help people interpret their experiences (Dweck, 2006), thus providing them with a meta-theoretical perspective to deal with tension (Schad et al., 2016). Drawing on the paradox literature, individuals might adopt a *dilemma* mindset or a paradox mindset to cope with tension. Individuals with a dilemma mindset may only perceive bedtime procrastination as a threat and opt for the “either/or” strategy, such as goal prioritization, which includes goal shelving (Mayer and Freund, 2022). This is because people prefer consistency in their beliefs and attitudes, and a dilemma mindset, which highlights contradictions, can cause anxiety. However, suppressing either leisure or sleep needs might exacerbate the negative consequences caused by resource depletion (Vince and Broussine, 1996).

In contrast, individuals with a paradox mindset tend to value, accept, and exist in harmony with tension. In other words, rather than viewing tension as opposing dichotomies or dilemmas that necessitate trade-offs, people with a paradox mindset view tension as opportunities to organize the complexities of reality and seek strategies to leverage them to uncover the beneficial outcomes (Miron-Spektor et al., 2018). People with a paradox mindset may manage tensions through *cognitive flexibility*, which broadens the scope of their attention span and allows them to consider divergent perspectives on an issue in a more balanced manner (Rothman and Melwani, 2017). A paradox mindset, like expanded cognition, would also foster *learning* (Smith and Tushman, 2005). The more distinctions people make about different perspectives or aspects of tension, the more they learn about the tension (or themselves), and the more likely they are to reach a point of convergence. For example, they may still retain some leisure time (and shorten it) before sleeping, but they will intentionally arrange more work breaks during the daytime to satisfy their leisure needs in a more balanced way. The paradox mindset might also enhance *recovery self-efficacy*—one's confidence in getting benefits from recovery time and opportunities (Sonnentag and Kruel, 2006). People with enhanced recovery self-efficacy are better at making use of leisure opportunities (e.g., during lunch or other micro-breaks) throughout the day (Trougakos and Hideg, 2009) and thus do not need to create more leisure opportunities by delaying their bedtime. Additionally, people with higher recovery self-efficacy have a larger resource repertoire, which means that a lack of sleep may have less of an impact on their fatigue level (Park and Sprung, 2015).

## 4. Discussion

Integrating research on paradox theory with bedtime procrastination, this work postulates that the paradox mindset is important for facilitating experiencing and responding to tension posed by bedtime procrastination, resulting in more constructive outcomes. Specifically, with a paradox mindset, people are more likely to embrace rather than avoid bedtime procrastination. Accepting both sides of a conflict increases the number of ideas and solutions on how to address it. Furthermore, a paradox mindset may enhance individuals' intrinsic motivation (Liu et al., 2020) to deal with bedtime procrastination behavior by engaging in agentic behaviors such as learning, goal-setting, and managing their tension in a more balanced way. In addition, people's recovery self-efficacy may also be improved.

Prior research suggests that mindsets can be learned and improved through training. For example, an earlier study found that the paradox mindset could be learned through living with tension (Lomranz and Benyamini, 2016). Intervention studies that have adopted paradoxical inquiry methods reported that managers can be taught to change their experience and approach to dealing with tension (Lüscher and Lewis, 2008). However, with limited empirical testing, it is unclear whether the improvement in mindset can be applied to the context of bedtime procrastination. To summarize, this opinion paper provides a novel approach to understanding and addressing bedtime procrastination. We hope this work can fuel and inspire future research aiming to address bedtime procrastination and other relevant health behaviors.

## Author contributions

The author confirms being the sole contributor of this work and has approved it for publication.
